# Impact of emergency medical support services on public health delivery system in Goa

**DOI:** 10.1186/1753-6561-6-S1-P14

**Published:** 2012-01-16

**Authors:** Priyanka Chaman

**Affiliations:** 1 Emergency Management and Research Institute, Vasant Kunj, New Delhi, India

## Introduction

As India is fighting to strengthen its health care delivery system, pre-hospital care (emergency ambulance services) still remains the most neglected part of India's healthcare service system. The importance of pre-hospital care is especially important in the rural areas where immediate health care is poor and services are distant. Most people in India succumb to death due to non-availability of quick and good quality emergency medical support.

Trauma continues to be one of the major causes of death in India. A report released by the transport research wing of the concerned ministry of the Indian government said that in 2008, the country witnessed 485,000 road accidents in which 120,000 people lost their lives. It is estimated that by 2020, trauma will become the third leading cause of deaths in India from its present position of ninth.

Maternal mortality also remains one of the most daunting public health problems in India and its reduction is a prominent component of the rural health programmes of the Indian government. India contributes approximately 20% to 24% of the world's maternal deaths. One of the '3 delays' identified for this is the delay in reaching an appropriate health care facility due to lack of affordable and accessible emergency transport.

A study conducted in Mpumalanga showed that lack of emergency transport between health institutions was a major factor in at least 38% of maternal mortalities recorded in the region. In such scenario, government of India identified the benefits of involving private players under the National Rural Health Mission (NRHM) in 'public-private partnership model'. The initial success of pre-hospital care services under Emergency Management and Research Institute (EMRI) has further boosted the confidence of policy makers. In this study, we present an assessment of the impact of EMRI services in the state of Goa based on all the emergencies reported in the months from September 2008 to March 2010.

## Methods

We used 'life saved data' from pre-hospital care records (PCR, which are emergency hospital care records maintained by the emergency medical technician in the ambulance), response records and 48-hour follow-up records maintained by the emergency response officer. A close-ended interview schedule was administered on 20 patients at the government hospital in Bambolim, Goa to understand the effectiveness of emergency response services of EMRI. We also reviewed literature on EMRI services in India.

## Results

We find an increase in utilisation of EMRI services. Of the total 43,835 patients handled by 108 emergency response services, 31.8% of the cases were trauma-related, 8.12% cardiovascular emergencies, and 7.5% pregnancy-related. Of the 50% of these patients that we followed up after 48 hours, we found that 3894 lives have been saved. The maximum lives saved were of trauma patients followed by cardiovascular emergencies. The services in the state averaged a response time of less than 30 minutes, the best response time for emergency services in all Indian states so far. A survey of the patients showed above-average satisfaction with the services in 95% of the patients.

## Discussion

EMRI has been able to instil confidence and trust among people especially in rural areas to use 108 ambulance services during medical emergencies as shown by the increased utilisation of and the satisfaction with the services. However government shall consider providing a continuous effective system of emergency medical care with a lead EMS (emergency medical service) agency nationwide, having the authority to plan appropriate rules and regulations for each recognised component of the EMS system such as standardised treatment, transport, communication and disaster management.

A comprehensive education survey needs to be conducted periodically to review the needs of the population and to ensure the providers have the skills and knowledge required to meet them.

